# RAFT Polymerisation and Hypercrosslinking Improve Crosslink Homogeneity and Surface Area of Styrene Based PolyHIPEs

**DOI:** 10.3390/polym15102255

**Published:** 2023-05-10

**Authors:** Amadeja Koler, Jiři Brus, Peter Krajnc

**Affiliations:** 1 PolyOrgLab, Faculty of Chemistry and Chemical Engineering, University of Maribor, Smetanova 17, 2000 Maribor, Slovenia; 2 Institute of Macromolecular Chemistry, Czech Academy of Sciences, Heyrovského náměstí 2, 16200 Prague, Czech Republic

**Keywords:** RAFT polymerisation, polyHIPEs, hypercrosslinking, porous polymers, porosity

## Abstract

The influence of a polymerisation mechanism (reversible addition–fragmentation chain transfer; RAFT vs. free radical polymerisation; FRP) on the porous structure of highly porous poly(styrene-co-divinylbenzene) polymers was investigated. The highly porous polymers were synthesised via high internal phase emulsion templating (polymerizing the continuous phase of a high internal phase emulsion), utilising either FRP or RAFT processes. Furthermore, residual vinyl groups in the polymer chains were used for the subsequent crosslinking (hypercrosslinking) applying di-tert-butyl peroxide as the source of radicals. A significant difference in the specific surface area of polymers prepared by FRP (between 20 and 35 m^2^/g) and samples prepared by RAFT polymerisation (between 60 and 150 m^2^/g) was found. Based on the results from gas adsorption and solid state NMR, it could be concluded that the RAFT polymerisation affects the homogeneous distribution of the crosslinks in the highly crosslinked styrene-co-divinylbenzene polymer network. During the initial crosslinking, RAFT polymerisation leads to the increase in mesopores with diameters between 2 and 20 nm, resulting in good accessibility of polymer chains during the hypercrosslinking reaction, which is reflected in increased microporosity. The fraction of micropores created during the hypercrosslinking of polymers prepared via RAFT is around 10% of the total pore volume, which is up to 10 times more than for polymers prepared by FRP. Specific surface area, mesopore surface area, and total pore volume after hypercrosslinking reach almost the same values, regardless of the initial crosslinking. The degree of hypercrosslinking was confirmed by determination of the remaining double bonds by solid-state NMR analysis.

## 1. Introduction

Porous materials are abundant in nature, especially porous polymer-based materials. Porous polymers can have high surface areas, have high pore volumes, and can contain modifiable functional groups for chemical modification, which makes them adaptable to specific applications [1].

An example of a porous polymer with tunable properties is a polyHIPE polymer, which is produced by the polymerisation of a high internal phase emulsion (HIPE) [2,3,4,5]. PolyHIPEs are synthesised by the polymerisation of the monomer (continuous) phase of the emulsion, which is dispersed in the internal (non-monomer) phase. The internal (non-monomer) phase occupies at least 64 vol.% of the total emulsion (random packing of droplets) [6]. The monomer phase of the emulsion polymerises during the polymerisation and forms the framework of the porous polymer, while the internal (non-monomer) phase serves as the pore template.

Most research on hydrophobic polyHIPEs was conducted on styrene-based systems wherein divinylbenzene was the most common crosslinker. In addition to the initial crosslinking of the polymers, they can be further crosslinked (hypercrosslinked) after the formation of the polymer network. Unlike crosslinking by the free radical polymerisation, polymer hypercrosslinking is a post-polymerisation process of crosslinking pre-existing polymer chains in a swollen state, which creates many new links or bridges between the existing chains [7,8]. During the hypercrosslinking the polymer chains are distributed throughout the polymer material and are strongly solvated. By hypercrosslinking a polymer gel, a microporous network is formed, while the hypercrosslinking of a macroporous polymer (polyHIPE) produces micropores in addition to the already existing macropores [9]. Such a bimodal pore distribution is highly desirable in many applications. Due to the formation of new micropores, hypercrosslinked polymers are characterised by high specific surfaces (up to 2000 m^2^/g) [10,11,12,13]. In addition to the high specific surface area, hypercrosslinked polymers retain their microporous structure even after the removal of the solvent, which generally does not apply to polymers with low crosslinking degrees. Therefore, the applicative possibilities of hypercrosslinked polymers are increased [14,15].

For the synthesis of polyHIPE materials, free radical polymerisation is generally used; however, it is limited in terms of monomer reactivity (double bonds) and the reaction conditions (high temperature). Controlled radical polymerisation represents an alternative to free radical polymerisation and enables the improvement of its shortcomings while also increasing the homogeneity of the polymer network. Reversible addition–fragmentation chain transfer (RAFT) polymerisation is a very useful controlled polymerisation for a wide range of monomers, with the possibility of obtaining polymers with predefined molecular weights, narrow polydispersities, and complex architectures [16,17,18,19].

RAFT polymerisation could also be used for the synthesis of polyHIPE materials, with the aim of increasing the control of the porous structure, improving the mechanical properties and the synthesis of necessary and well-defined functional groups on the surface of the pores.

An example of utilising RAFT polymerisation in the preparation of polyHIPEs was shown through the preparation of an amphiphilic macro RAFT reagent (block copolymer of styrene and acrylic acid) as a surfactant to stabilise w/o and o/w HIP emulsions [20]. By using RAFT polymerisation it was possible to prepare a block copolymer with good control of the monomer ratio units in the block copolymer chains, therefore enabling the synthesis of an appropriate block copolymer for the stabilisation of a monomer mixture consisting of styrene and acrylic acid and the consequent polymerisation of the HIPE into a polyHIPE, forming poly(styrene-co-acrylic acid) polyHIPE materials. Additionally, the functional groups of the macro RAFT surfactant were used for subsequent surface modification of the synthesised polyHIPE. Similarly, use of RAFT reagent with amphiphilic character, based on acrylate/acrylic acid blocks [21] or poly(ethylene glycol) methyl ether acrylate [22], were used as the emulsion stabiliser. RAFT polymerisation was also used to graft N-isopropyl acrylamide [23] and glycidyl methacrylate [24] from the polymer surface. In the case of NIPAAm, the polyHIPE was based on primary amine, while in the case of GMA, a hypercrosslinked poly(VBC/DVB) polyHIPE was used.

Only a few examples of the direct use of RAFT polymerisation for the synthesis of polyHIPE materials were reported so far. Poly(STY/DVB) polyHIPE was synthesised in the presence of various RAFT reagents, which can control the course of RAFT polymerisation [25]. The results show that the Young’s modulus and the crush strength of RAFT poly(STY/DVB) polyHIPE materials increased four times compared to polyHIPEs prepared by free radical polymerisation. The results of the aforementioned study indicate greater homogeneity of the polymer walls as a result of the use of controlled RAFT polymerisation. In other research, the influence of the polymerisation mechanism (RAFT and FRP), RAFT reagent to initiator ratio, initiator solubility and crossliker content upon the porous structure, the mechanical properties, and the swelling of poly (STY/DVB) polyHIPEs were studied [26]. Differences in pore size and pore connectivity, mechanical and thermal properties, and uptake behavior of polymers synthesised by RAFT and FRP were again revealed and are reflected in the increased mobility and uniformity of macromolecules when using RAFT polymerisation. Another report describes the polymethyl methacrylate-based polyHIPEs using the RAFT mechanism [27]. In spite of the advantages of RAFT polymerisation, it is still rarely used for the synthesis of polyHIPE materials (so far, only the above-mentioned syntheses are known), as there is still a lack of understanding of the crosslinking mechanism within RAFT polymerisation [28].

The aim of this work was to study the affect of the polymerisation mechanism (RAFT vs. FRP) and crosslinker content on the porosity features and responsiveness to hypercrosslinking of styrene-based polyHIPEs.

## 2. Experimental

### 2.1. Materials

Styrene (STY, Sigma Aldrich, Darmstadt, Germany) and divinylbenzene (DVB, technical grade, containing 80% divinylbenzene and 20% ethyl vinyl benzene, Sigma Aldrich, Darmstadt, Germany) were purified by filtration through basic alumina (Al_2_O_3_, Fischer Chemical, Pittsburgh, PA, USA) to remove the inhibitors.

Sorbitan monooleate (Span 80, Sigma Aldrich, Darmstadt, Germany), α,α’-azo-bisisobutyronitrile (AIBN, Fluka, Buchs, Switzerland), acetone (Sigma Aldrich, Darmstadt, Germany), toluene (Sigma Aldrich, Darmstadt, Germany), di-tert-butyl peroxide (DTBP, Luperox DI, 98%, Merck, Darmstadt, Germany), calcium dichloride hexahydrate (CaCl_2_ × 6H_2_O, 98%, Sigma Aldrich, Darmstadt, Germany), and 2-(dodecylthiocarbonylthio)-2-methylpropanoic acid (DDMAT, Merck, Darmstadt, Germany) were used as received.

### 2.2. Synthesis of STY/DVB PolyHIPE Materials by Free Radical Polymerisation

**Scheme 1:**
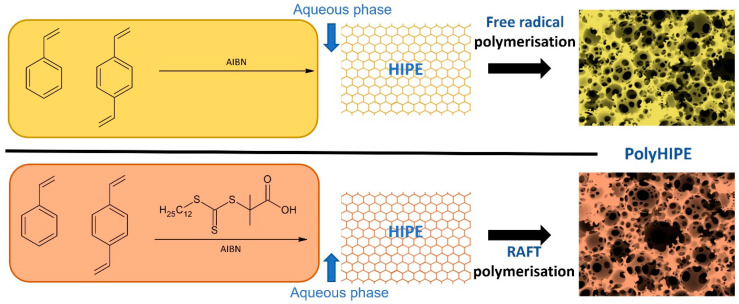
Synthetic procedure for polyHIPEs preparation.

The continuous phase, consisting of monomers STY and DVB, the surfactant Span 80 (20 wt.% to monomers), and the initiator AIBN (1 wt.% to monomers), was placed in a two-necked reactor, to which the degassed aqueous phase (80 vol.% of the emulsion, 1.76% aqueous solution of CaCl_2_) was added using a dropping funnel at constant stirring (see Table 1 for amounts). Following the addition of the aqueous phase, the emulsion was stirred with an overhead stirrer for 1 h at 300 rpm. The emulsion was transferred to a polypropylene mould and polymerised in an oven for 24 h at 60 °C. The monoliths were extracted in a Soxhlet apparatus with water (24 h) and acetone (24 h) and then air-dried. Samples prepared by free radical polymerisation were labelled as **FRP**. See Scheme 1 for a schematic presentation of the synthetic procedure.

### 2.3. Synthesis of STY/DVB PolyHIPE Materials by RAFT Polymerisation

RAFT polymerisation of STY/DVB polyHIPE materials was carried out in the same way as the free radical polymerisation of STY/DVB polyHIPES, except that 2-(dodecylthiocarbonothioylthio)-2-methylpropanoic acid DDMAT was added to the continuous phase and purged with nitrogen throughout the HIPE formation in a different monomer-to-initiator ratio (Table 2). The polymerisation was carried out for 24 h at 60 °C and the resulting monoliths were washed with water and acetone (for 24 h in each solvent) by Soxhlet extraction and then air-dried. Samples prepared by RAFT polymerisation were labelled as **RAFT**.

### 2.4. Hypercrosslinking Procedure

A total of 1 g of the grinded sample and 20 mL of toluene were placed into a flask and stirred for two hours to swell the polymer. The radical initiator di-tert-butyl peroxide DTBP (10 w. % by weight of polymer) was then added, and the mixture was heated for 20 h under reflux. The polymers were filtered and washed with tetrahydrofurane (3 × 50 mL) and air-dried.

The samples were labelled in the same way as the STY/DVB polyHIPEs, with the addition of H (for hypercrosslinked).

### 2.5. Characterisation

The specific surface area of the polymers was determined by measuring the adsorption/desorption of nitrogen via the BET method, the BJH method was used for the pore size distribution, and the t-plot analysis was performed to determine the volume of micropores present. For this purpose, the Micromeritics TriStar II 3020 instrument (Micromeritics, Norcross, GA, USA) was used with measurements performed at 77.4 K. All samples were purged with nitrogen (24 h at 40 °C) prior to analysis.

Porous morphology was studied by scanning electron microscopy (SEM). SEM images were taken with a Phillips XL-30 SEM scanning electron microscope at 20 kV. The samples were sputtered with platinum on a Quorum Q150R E for 3 min at 40 mA. The sizes of the primary pores were determined using SEM images by measuring at least 50 pores, taking into account a correction factor of 2/√3 [29].

Solid-state NMR spectra were measured at 11.7 T using a Bruker Avance 500 WB/US NMR spectrometer (Karlsruhe, Germany, 2013) in a 3.2 mm double resonance probehead. The combined technique of single-pulse and cross-polarisation excitation of the ^13^C magnetisation was used to record the ^13^C SP/CP/MAS NMR spectra. This experiment was used to detect both rigid and mobile components in a single spectrum. The magic angle spinning frequency was set to 20 kHz (MAS); the recycle delay was 10 s and the cross-polarisation contact time was 1 ms. The strength of the *B*_1_(^13^C) spin-locking fields, expressed in frequency units *ω*_1_/2π = γ*B*_1_, was 64 kHz. The number of FID accumulations was 10,128. The spectra were referenced to *α*-glycine (176.03 ppm). Frictional heating [30,31] of the spinning samples was compensated by active cooling and temperature calibrations were performed with Pb(NO_3_)_2_.

## 3. Results and Discussion

### 3.1. PolyHIPE Preparation: FRP vs. RAFT

In this work, highly porous (80% nominal porosity) and highly crosslinked (using 50, 60, and 70 mol% of DVB) poly (styrene-co-divinylbenzene) polyHIPEs were prepared by either FRP or RAFT polymerisation. A high crosslinker content was used due to the ability to use the unconsumed vinyl groups in the post-polymerisation hypercrosslinking process, and typically, the high initial crosslinker content is a requirement for this. With a low crosslinking degree, most vinyl groups find reaction partners in suitable position and distance during the initial polymerisation to form a crosslinked polymer chain. As a result, such polymers are less suitable for further post-polymerisation hypercrosslinking [32]. Unlike polyHIPEs synthesised by free radical polymerisation, RAFT polymerisation was performed in the presence of a suitable RAFT reagent, which allows for more control of the propagation process. For this purpose, the 2-(dodecylthiocarbonothioylthio)-2-methylpropanoic acid RAFT reagent [33,34,35] was added to the organic phase in addition to monomers, Span 80 surfactant, and the AIBN initiator. In the RAFT polymerisation of polyHIPE materials RAFT1-3, a relatively high initiator/RAFT reagent (2.8) ratio was used. A high ratio should affect the formation of short chains and increase the possibility of termination reactions, but for highly crosslinked polymers, this effect should not be significant.

**Figure 1:**
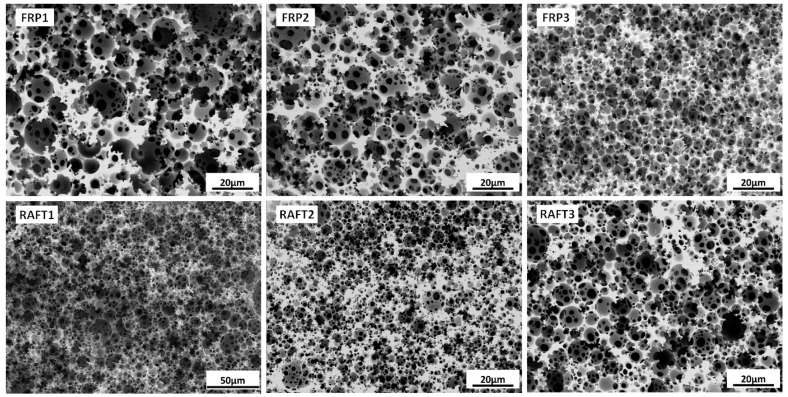
SEM images of FRP1-3 and RAFT1-3 samples.

The morphological properties of FRP and RAFT polyHIPE materials were characterised by scanning electron microscopy (Figure 1). All polymers exhibit an interconnecting open cellular polyHIPE structure with pores between 5 and 12 µm in diameter. The sizes of the macropores do not differ significantly depending on the type of polymerisation, only in the case of 60% crosslinking is there a greater difference. This suggests that the emulsion stability was not significantly affected by the choice of initiator and polymerisation process. The pore size in the FRP2 sample was measured to be 12 µm, while the pore size in the RAFT2 sample was 5 µm. The pore sizes of 50% crosslinked polyHIPE polymers were measured to be around 10 µm and 7 µm for 70% crosslinked.

All prepared samples were investigated by nitrogen adsorption/desorption porosimetry, applying the BET model for surface area determination, BJH method for evaluation of mesopore size distribution, and t-plot for micropore volume assessment. The BET specific surface area of FRP samples was determined to be between 19 and 35 m^2^/g, while for RAFT samples, significantly higher values were obtained, namely between 60 and 156 m^2^/g (Table 3).

**Figure 2:**
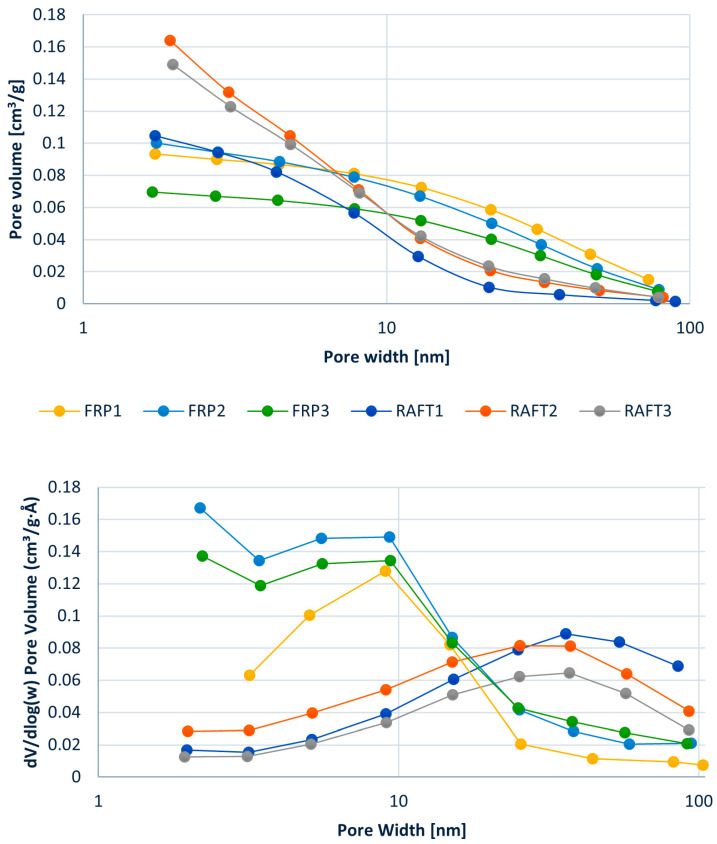
Mesopore size distribution in FRP and RAFT samples (BJH method).

It can be seen from the nitrogen adsorption/desorption isotherms that the synthesised materials lack micropores. The increase in specific surface area in RAFT samples is due to the formation of a larger number of mesopores. The size distribution of mesopores (BJH model) shows a clear difference between the FRP and RAFT samples (Figure 2). In RAFT samples, the maximum occurrence of pores is from 2 to 20 nm, while in FRP samples, only pores larger than 20 nm are present, suggesting that the RAFT polymerisation has an impact on the porous structure in the range of mesopores. During the RAFT polymerisation, the crosslinks are more homogeneously distributed throughout the polymer network, which leads to the formation of predominantly mesopores. In the case of FRP, in the course of crosslinking, micrometer gels are formed, which leads to the formation of a heterogeneous crosslinked network, and consequently, to the formation of pores larger than 20 nm. This is reflected in the significant difference in surface areas found when comparing FRP and RAFT samples.

### 3.2. Post-Polymerisation Crosslinking (Hypercrosslinking)

Poly(STY/DVB) polyHIPEs were used for a subsequent crosslinking reaction with di-tert-butyl peroxide. In the case of high initial crosslinking (high DVB content), not all vinyl groups can find a partner for radical polymerisation due to position or distance restrictions, and thus a number of vinyl groups remain unreacted [36]. After the polymerisation, the remaining vinyl groups can be activated with di-tert-butyl-peroxide, which is capable of abstraction of hydrogen atoms from aliphatic carbons, thus forming the active centers [32]. An active radical can react with a nearby unreacted double bond, and thus crosslink the polymer chains.

Unreacted double bonds of FRP and RAFT polyHIPE materials were subsequently hypercrosslinked with the radical initiator di-tert-butyl-peroxide, with the aim of observing whether RAFT polymerisation affects the hypercrosslinking reaction. Hypercrosslinking with a radical initiator is a known procedure that was used before [37].

**Figure 3:**
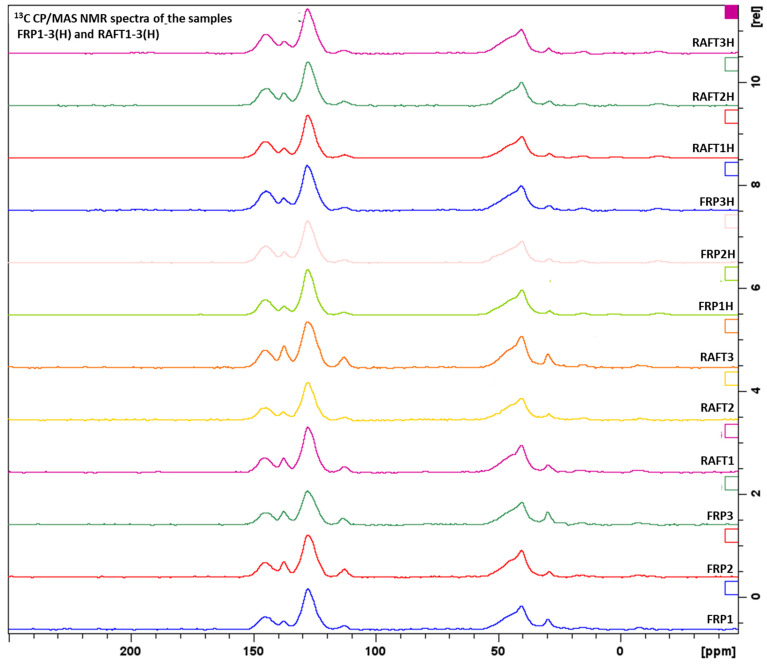
^13^C SP/CP/MAS NMR spectra of the samples FRP1-3(H) and RAFT1-3(H) at 20 kHz.

In order to study the concentration of unreacted vinyl groups before and after post-polymerisation crosslinking, ^13^C ss-NMR spectroscopy was used. The ^13^C NMR signals recorded in the ^13^C SP/CP/MAS NMR spectra of FRP(H) and RAFT(H) materials (Figure 3) reflect typical DVB/ethylstyrene systems. The narrow signals resonating between 30 and 10 ppm represent mobile terminal CH_2_ and CH_3_ units. At 112 and 137 ppm, we found clear signals for unreacted double bounds reflecting CH_2_= and CH= carbon atoms, respectively. The two signals at 146 and 128 ppm represent six carbon atoms of both styrene and DVB monomer units. Comparing the ^13^C NMR spectra of polymers before and after hypercrosslinking, no significant changes were observed.

Subsequently, molar amounts of residual double bonds were determined by comparison of the integrals of the remaining vinyl groups at 112 ppm (CH_2_=), with the integrals of aromatic carbons resonating at 146 and 128 ppm. This way, the molar amount of residual double bonds per average monomer unit was determined.

It can be seen from Table 4 that the highly crosslinked polyHIPE materials (before hypercrosslinking) have a rather high proportion of unreacted double bonds (between 29 and 45 mol. %), while the mol. % of unreacted double bonds decreases after additional radical reaction with di-tert-butyl-peroxide (between 24 and 30 mol. %) in all poly(STY/DVB) polyHIPEs. The decrease in mol. % of double bonds after subsequent crosslinking confirms the process of the hypercrosslinking reaction. Hypercrosslinking took place in both FRP and RAFT samples, as the mol. % of remaining double bonds decreases in all samples regardless of the type of polymerisation.

Table 4 shows that the mol. % of residual double bonds (before hypercrosslinking) in the poly(STY/DVB) polyHIPE materials prepared by free radical polymerisation does not drastically differ from the materials prepared by RAFT polymerisation. For 50% (FRP1 and RAFT1) and 70% (FRP3 and RAFT3) crosslinked polymers, there are about 3 mol. % more free double bonds in RAFT polymers, while a greater deviation is observed for 60% crosslinked materials, namely, in the sample FRP2, there are about 10 mol. % more free double bonds than in the sample RAFT2. According to the results of ^13^C ssNMR analysis, it can be concluded that the type of polymerisation does not have a drastic effect on the consumption of double bonds during the post-polymerisation hypercrosslinking.

**Figure 4:**
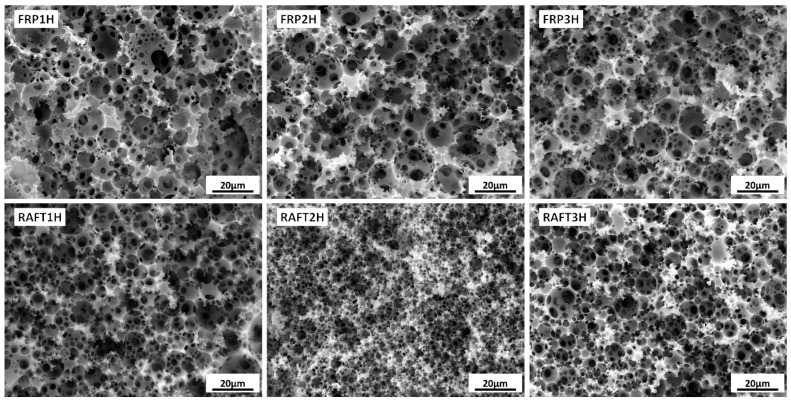
SEM images of FRP1-3H and RAFT1-3H samples.

The data from the BET model of specific surface area show an increase in the specific surface area after hypercrosslinking in all prepared polymers (40–290 m^2^/g) (Table 5), which indicates the introduction of new porosity into the material. Despite the induction of new porosity, the macroporous open cell polyHIPE structure remains unchanged after subsequent crosslinking (see Figure 4 for SEM images).

The substantial increase in the BET specific surface area indicates the formation of new micropores during the hypercrosslinking reaction in all samples. The increase in the specific surface area is due to the creation of many new connections, which are formed due to the additional post-polymerisation crosslinking of unreacted double bonds, which are sufficiently flexible and proximate to be able to react with each other. However, the BET surface area model implementation is not the most suitable method to get a real insight into the results when dealing with microporous polymers [38]. More relevant conclusions regarding microporosity can be obtained using the t-plot method, which determines the volume of micropores and the external surface area (mesopore surface area) of the samples. A lack of micropores was observed in samples before hypercrosslinking, shown by zero or slightly negative micropore volume as determined by the t-plot analysis. After hypercrosslinking, the micropore volume increases for both FRP (0.0011–0.0105 cm^3^/g) and RAFT hypercrosslinked samples (0.0205–0.0246 cm^3^/g).

**Figure 5:**
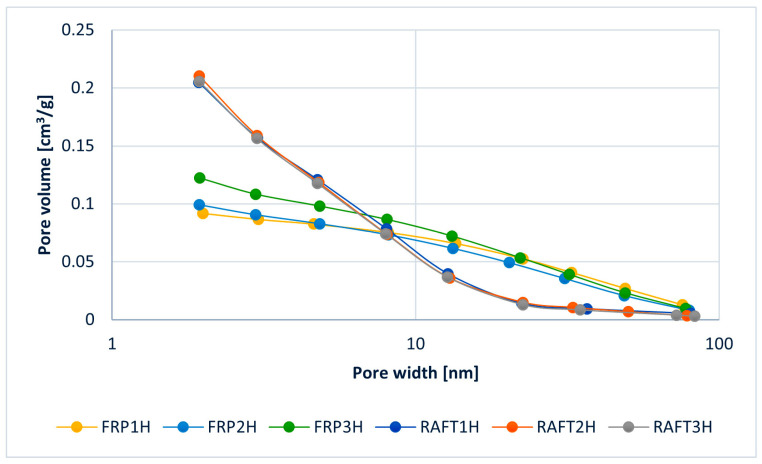
Mesopore size distribution in hypercrosslinked FRP and RAFT samples (BJH method).

The fraction of micropores formed during the hypercrosslinking of FRP samples varies with crosslinker content. Less crosslinked FRP samples have a lower fraction of micropores formed and vice versa (1% fraction of micropores (at 50 mol. % DVB), 5% (at 60 mol. % DVB) and 8 mol. % (70% DVB) (Table 5)). It can be concluded that with lower initial crosslinking (FRP1), the unreacted double bonds are further apart than in more crosslinked polymers (FRP2 and FRP3); and thus, there is less possibility for the formation of new connections between them, and therefore less probability for the formation of new micropores. In the case of RAFT hypercrosslinked samples (regardless of the initial crosslinking), almost no differences are noticeable in the measurements for BET specific surface area, external (meso) surface area, micropore volume, and the proportion of newly formed micropores after the hypercrosslinking (Table 5). Micropores created in RAFT hypercrosslinked samples represent approximately 10% of the total pore volume, which is up to 10 times more than in FRP samples. The mesopore size distribution of hypercrosslinked FRP and RAFT materials show that RAFT hypercrosslinked samples have a significantly higher number of mesopores up to 5 nm in diameter (Figure 5).

RAFT polymerisation within HIPEs affects the formation of a porous structure due to a more uniform distribution of crosslinks and controlled chain growth; therefore, more flexible chains are formed, which results in homogeneously distributed crosslinks in the polymer network regardless of the initial crosslinking. The porosity (porous profile), especially in the meso and micropore domain, is strongly affected by the crosslink density and by the regularity of crosslinks (their regional distribution), and this is the main reason for the differences in the mesopore region observed. As the surface area is much more affected by the mesopore domains than by the macropore domains (which do not depend significantly on the polymerisation mechanism, but rather on the stability of the high internal phase emulsion), the difference in porous structure is relevant. Due to the homogeneity of the initially crosslinked RAFT polymer network, the entire polymer material is more accessible to reagents, which is reflected in the similarity of the results obtained with the nitrogen adsorption/desorption experiment after the hypercrosslinking (Table 5). In the swollen state, the radical initiator di-tert-butil-peroxide for subsequent crosslinking can be distributed throughout the polymer. The surface of the polymer is well accessible to the initiator, and the polymer chains are flexible and close enough for additional crosslinking to take place. Due to the uneven distribution of crosslinks, polymers prepared by FRP have more or less crosslinked domains, which are not equally accessible.

## 4. Conclusions

RAFT polymerisation affects the course of the crosslinking of poly(STY/DVB) polyHIPE samples, which is shown in a significantly higher number of formed mesopores, subsequently increasing the BET specific surface area (up to 150 m^2^/g), compared to polymers prepared by free radical polymerisation (up to 35 m^2^/g). This is due to the more uniform distribution of the crosslinks in the RAFT polymerised samples, which causes an increase in the formation of mesopores compared to the polymers prepared by free radical polymerisation. Additionally, the initial polymer structure of the RAFT crosslinked polymers changes the porous structure formed after the hypercrosslinking, resulting in the RAFT polymer having a higher degree of microporosity. In comparison, the proportion of the micropores in FRP samples depends on the initial content of the crosslinker (increases with increasing DVB). It needs to be noted that the type of polymerisation (RAFT vs. FRP) did not significantly affect the content of the unreacted double bonds before and after the hypercrosslinking, suggesting that the reactivity of the monomers is approximately the same. Considering the homogenous distribution of the crosslinks in the polymer network, RAFT polymers are uniformly accessible throughout the material, which was demonstrated by the results of the BET specific surface area, the external (meso) surface area, the fraction of the micropores, and the total pore volume of the hypercrosslinked RAFT polyHIPEs. Regardless of the crosslinker content in RAFT non-hypercrosslinked polyHIPEs, a plateau is reached after the hypercrosslinking with similar values for the volume and specific surface areas of micro and mesopores.

## Figures and Tables

**Table 1:** Composition of polyHIPE samples using free radical polymerisation.

Sample	m (STY) [g]	m (DVB) [g]	V_WP_ [mL]	X_xlink_
**FRP1**	2.114	4.479	40.0	50
**FRP2**	1.098	3.956	30.7	60
**FRP3**	0.761	6.114	42.0	70

V_WP_ volume of aqueous phase; X_xlink_ mol% of DVB.

**Table 2:** Composition of polyHIPE samples using RAFT polymerisation.

Sample	m (STY) [g]	m (DVB) [g]	m (RAFT) [g]	m (AIBN) [g]	V_WP_ [mL]	X_xlink_
**RAFT1**	2.061	4.396	0.051	0.064	40.0	50
**RAFT2**	1.128	3.937	0.045	0.049	30.7	60
**RAFT3**	0.707	6.128	0.054	0.068	42.0	70

V_W_ volume of water phase; X_xlink_ mol% of DVB.

**Table 3:** BET surface area of FRP and RAFT samples.

Sample	Crosslinker Content (mol%)	BET Surface Area [m^2^/g]	V_pore_ [cm^3^/g]	V_micro_ * [cm^3^/g]	Micropore Area [m^2^/g] *	External Surface * [m^2^/g]
**FRP1**	50	24.6	0.089	/	/	30.3
**FRP2**	60	35.4	0.095	/	/	43.1
**FRP3**	70	19.4	0.066	/	/	24.3
**RAFT1**	50	60.9	0.100	/	/	73.2
**RAFT2**	60	156.5	0.166	/	/	152.3
**RAFT3**	70	108.8	0.138	/	/	120.5

* results obtained from t-plot.

**Table 4:** Molar amounts of residual double bonds (–CH=CH_2_) per monomer unit with aromatic ring determined from ^13^C CP/MAS NMR spectra of the investigated FRP and RAFT systems before and after hypercrosslinking.

Sample	Crosslinker Content (mol%)	mol. % (C=C)	mol. % (C=C) ^HP^
**FRP1**	50	29.14	25.00
**FRP2**	60	39.00	26.07
**FRP3**	70	42.20	25.78
**RAFT1**	50	33.60	24.67
**RAFT2**	60	29.76	26.48
**RAFT3**	70	45.25	30.65

^HP^ after hypercrosslinking.

**Table 5:** BET surface area, micro and mesoporosity of FRPH and RAFTH samples.

Sample	Initial Crosslinker Content (mol%)	BET Surface Area [m^2^/g]	V_pore_ [cm^3^/g]	V_mikro_ * [cm^3^/g]	V_mikro_/V_pore_	V_meso_ * [cm^3^/g]	Micropore Area [m^2^/g] *	External Surface * [m^2^/g]
**FRP1H**	50	40.8	0.095	0.0011	0.012	0.0939	3.3	37.5
**FRP2H**	60	90.3	0.124	0.0066	0.053	0.1174	16.2	74.1
**FRP3H**	70	94.2	0.126	0.0105	0.083	0.1155	24.6	69.6
**RAFT1H**	50	273.3	0.236	0.0205	0.087	0.2155	49.9	223.2
**RAFT2H**	60	289.7	0.244	0.0246	0.101	0.2194	60.1	229.7
**RAFT3H**	70	282.4	0.239	0.0220	0.092	0.2170	53.4	228.9

* results obtained from t-plot.

## Data Availability

The data that support the findings of this study are available from the corresponding author upon reasonable request.
